# Supplementary material for: Analysis and prediction of carbon storage changes on the Qinghai-Tibet Plateau
Source: PLoS One. 2025 Apr 7;20(4):e0320090. doi: 10.1371/journal.pone.0320090 (PMC11975099; doi:10.1371/journal.pone.0320090)
Supplement: Attachment — Submitted filename: Response.pdf [file pone.0320090.s001.pdf]

Dear Reviewers,

On behalf of all contributing authors, I would like to express our sincere appreciation for the reviewers' constructive comments on our article entitled "Analysis and Prediction of Carbon Storage Changes on the Qinghai-Tibet Plateau" (Manuscript No.: PONE-D-24-41312). We are grateful for the reviewers' valuable and helpful feedback on our manuscript. We have carefully reviewed the comments and made revisions accordingly. Based on the instructions provided in your letter, we have revised the manuscript, with modifications highlighted in yellow and blue—yellow indicating new content and blue indicating more standardized English expressions. Point-by-point responses to the and reviewers' comments are provided below this letter.

We would like to express our gratitude for the opportunity to resubmit a revised version of the manuscript and for your time and consideration.

We look forward to hearing from you soon.

Yours sincerely,

Yaping Zhang

Reviewer1:

Comments:

The Qinghai-Tibet Plateau, as a crucial global carbon reservoir, plays a pivotal role in the carbon cycle. Studying changes in its carbon storage is of great significance for understanding global climate change. The paper uses the InVEST model and PLUS model to analyze changes in carbon storage on the Qinghai-Tibet Plateau and predicts future trends, demonstrating certain innovation and practicality in this research area. However, there is still room for improvement in research methods and results presentation, yet the paper provides certain reference value for studying carbon storage changes on the Qinghai-Tibet Plateau. There are still several issues that need to be addressed, particularly the inadequate discussion.

Reply: The authors want to thank the reviewer for helpful comments. The authors have carefully revised these shortcomings in the manuscript.

1. The quality of Figure 1 needs improvement.

Reply 1: Thanks for the reviewer's nice comment. The authors adjusted the colors in Figure 1 and revised the annotations for the regions, using abbreviations instead of full names to save unnecessary space and enhance the image's visual appeal and readability.

2. Tables 2-4 could be merged into a single table or figure for better clarity.

Reply 2: Thanks for the reviewer's excellent comments. The authors have merged tables 2 to 4 into a new Table 2, improving clarity while reducing unnecessary space usage. The new table is placed in section 2.3 on page 5.

3. The number of cited references throughout the paper is too low, representing a notable omission in this aspect.

Reply 3: Thanks for the reviewer's helpful comments. The authors have added relevant references in appropriate sections of the paper. These include works for comparison with our conclusions, papers supporting certain statements in the text, and references related to the selection and use of data.The newly added or modified reference numbers by the authors are 31-35, 39-40, and 42-69.

4. *The simulated data lacks comparison with other studies, and the parameters in Table 1 do not address vegetation and climate zoning.*

Reply 4: The authors thank the reviewer for their valuable comments. In response to the reviewer's suggestions, we have conducted a detailed evaluation and analysis of the simulated data, comparing it with data from related studies, and have revised the discussion section accordingly. We have added Section 4.1 (on page 14) to assess the carbon storage simulation data. Additionally, in Sections 4.2 to 4.4 (formerly Sections 4.1 to 4.3), we further analyze the impact of various driving factors on carbon storage by comparing our results with those of other studies.

5. The discussion section needs to be compared with previous research findings, which is the biggest issue of this paper. It tends to present the results in isolation without sufficient reference to existing literature. The discussion section is severely lacking in relevant references and needs to be reorganized.

Reply 5: The authors sincerely thank the reviewer for the valuable comments. In response, we have thoroughly revised and reorganized the discussion section to better compare and contextualize our findings with previous research. Relevant conclusions have been analyzed in relation to existing literature, and modifications have been made throughout Sections 4.1 to 4.7. The specific modifications have been highlighted in the text.

Reviewer2:

1. Gaps and research innovations to be filled by research need to be further clarified.

Reply 1: The authors appreciate the reviewer's valuable comments. Following the reviewer's suggestions, we have reorganized the introduction to provide a more detailed discussion on the importance of studying carbon storage changes and their driving factors, as well as the limitations of existing research. Additionally, the authors have summarized the contributions and research objectives of this study, emphasizing how it addresses the shortcomings of current methods. We revised the wording in the second paragraph of the introduction on page 2, merged and modified the third and fourth paragraphs, and summarized the contributions and research objectives in the final paragraph of the introduction.

2. Explain why these variables and models were chosen for the uniqueness of the Tibetan Plateau.

Reply 2: The authors want to thank the reviewer for the nice comments. In terms of variable selection, the ecosystem of the Qinghai-Tibet Plateau is highly fragile, impacted by climate change (e.g., rising temperatures, changes in precipitation, and increasing aridity) and human activities (e.g., overgrazing and industrial development). This vulnerability makes ecological restoration especially crucial. Therefore, selecting drivers related to ecosystem health—such as soil properties and vegetation cover—can help researchers identify key factors influencing restoration, facilitating the development of more effective restoration strategies. Furthermore, the ecosystem services of the Qinghai-Tibet Plateau—such as water conservation, soil retention, and eco-tourism—are essential for regional sustainable development. By incorporating variables linked to ecosystem services, such as proximity to railways or roads, researchers can comprehensively evaluate the enhancement of ecological service value when assessing the cost-effectiveness of restoration projects, providing scientific support for restoration efforts. The plateau's complex topography, with significant slope variability, further exacerbates the impact of slope aspects. Therefore, including topographic factors like slope and aspect in the analysis is essential.

Regarding model selection, the high altitude and low temperatures of the Qinghai-Tibet Plateau make large-scale ground surveys costly and labor-intensive. On the other hand, remote sensing lacks the precision of ground-level details, posing challenges to accurately assessing carbon storage in the region. Model simulations offer a cost-effective solution, providing broader spatial and temporal coverage, integrating multi-source data, and supporting complex decision-making analyses.

Compared to other models, the PLUS (Patch-generating Land Use Simulation) model is particularly suited for handling complex land-use changes. It combines multiple random seeds with cellular automata (CA), offering higher simulation accuracy. This is especially important for ecologically diverse regions like the Qinghai-Tibet Plateau, which face multiple pressures. The model can reveal the driving mechanisms behind land-use changes, aiding in the formulation of more precise restoration strategies. Additionally, given the Qinghai-Tibet Plateau's role as a significant global carbon sink,

the selection of the InVEST model for carbon storage assessment highlights its considerable ecological value and the critical role of carbon storage in climate regulation and ecosystem stability, enhancing our understanding of ecological potential and challenges under future scenarios. OLS fitting can provide an overall trend analysis, but given the complexity of the Qinghai-Tibet Plateau's geography, as well as its significant climate and ecosystem diversity, OLS is insufficient to capture local variations. Different regions' ecosystems respond uniquely to climate, topography, and human activities, resulting in diverse impacts across areas. GWR, on the other hand, assigns different regression coefficients to each observation point spatially, allowing the analysis to reflect localized characteristics for different regions. This approach is well-suited to the Qinghai-Tibet Plateau, where geographic and climate conditions are complex and vary significantly across regions. GWR can reveal which driving factors, such as altitude, temperature, or NDVI, have a more substantial impact on carbon storage or ecosystem services in specific areas, providing a more refined basis for management.

The authors have added explanations in Section 2.2 (Dataset) on page 4 to clarify the reasons for selecting relevant variables. Additionally, further details on model selection have been included in Sections 2.3.2 to 2.3.4.

The authors have added explanations in Section 2.2 (Dataset) on page 4 to clarify the reasons for selecting the relevant variables.

The description of the PLUS model in Section 2.3.2 on page 5 has been revised to clarify the specific reasons for selecting this model.

Similarly, the description of the InVEST model in Section 2.3.3 on page 6 has been revised to explain the rationale for selecting this model, with the specific modifications highlighted in the text.

In Section 2.3.4 on page 8, the authors have added explanations for selecting the OLS and GWR models to conduct relevant research on the Qinghai-Tibet Plateau.

Due to the limited research on land use prediction for the Tibetan Plateau and the variability introduced by different land use datasets, there may be some discrepancies when comparing simulation data with other studies. The land use data in this study is based on the Chinese annual land cover dataset (CLCD) created by Professor Huang Xin at Wuhan University using Landsat data, with an overall accuracy of 80%. Through model accuracy validation, it can be demonstrated that the future land use data simulated by the PLUS model is reliable. The authors added content regarding model validation in Section 2.3.2 on page 6.

3. Use more spatial distribution and trend plots to enhance the readability of the results section.

Reply 3: The authors want to thank the reviewer for the useful comments. The authors added a land use change figure (Fig. 8), illustrating the transition relationships between various land use types from 2020 to 2040, in Section 3.3.1 on page 12. The specific modifications have been highlighted in the text.

The authors added a total carbon storage change figure (Fig. 10), illustrating the changes in total carbon storage on the Qinghai-Tibet Plateau from 2020 to 2040, in Section 3.3.2 on page 14. The specific modifications have been highlighted in the text.

4. The heterogeneity of factors across regions needs to be clarified in terms of how carbon management policies can contribute to ecological conservation.

Reply 4: The authors want to thank the reviewer for helpful comments. The authors reorganized the section 4.7 (page 21) on future scenario predictions and management strategies to better reflect the impact of different regional driving factors on carbon storage in the Qinghai-Tibet Plateau, thereby providing targeted and regionally heterogeneous policy recommendations. The specific modifications have been highlighted in the text.
