# Supplementary material for: Analysis and prediction of carbon storage changes on the Qinghai-Tibet Plateau
Source: PLoS One. 2025 Apr 7;20(4):e0320090. doi: 10.1371/journal.pone.0320090 (PMC11975099; doi:10.1371/journal.pone.0320090)
Supplement: Attachment — Submitted filename: Response_auresp_2.pdf [file pone.0320090.s002.pdf]

Dear reviewer:

On behalf of all the authors, I would like to express my sincere gratitude to the reviewers for their affirmation of our article titled "Analysis and Prediction of Carbon Stock Changes in the Qinghai Tibet Plateau" (manuscript number: PONE-D-24-41312R1). We greatly appreciate the valuable and beneficial feedback from the reviewers on our manuscript. According to the instructions in your letter, we have revised the manuscript and highlighted the modified parts in yellow and blue - yellow represents new content and blue represents more standard English expression. Below this letter is a point by point response to the reviewer's comments.

We greatly appreciate the opportunity to resubmit the revised manuscript and thank you for your time and consideration.

We look forward to hearing from you soon.

Yours sincerely,

Yaping Zhang

Editor:

Please review your reference list to ensure that it is complete and correct. If you have cited papers that have been retracted, please include the rationale for doing so in the manuscript text, or remove these references and replace them with relevant current references. Any changes to the reference list should be mentioned in the rebuttal letter that accompanies your revised manuscript. If you need to cite a retracted article, indicate the article's retracted status in the References list and also include a citation and full reference for the retraction notice.

Reply: The authors want to thank the editor for their important suggestions. The authors checked the references and made modifications to some of them. [1] is a special report with an incorrect citation format, and the authors have replaced it with an article.

1. Kerr, Richard A. Global warming is changing the world. American Association for the Advancement of Science. 2007;316(5822):188–190.

[6] is an online article, the authors have modified the format of the references.

6. Nixon J. The economic impact of global warming. An Oxford Economics White Paper. 2020. Available from:
https://www.cheshirescouts.org.uk/wp-content/uploads/The-economic-impact
of-global-warming-Oxford-Economics.pdf

The author information abbreviation in reference [11] is incorrect, and the authors have corrected it.

11. Chen GS, Tian HQ. Land use/cover change effects on carbon cycling in terrestrial ecosystems. Chinese Journal of Plant Ecology. 2007;31(2):189.

The abbreviation of the publishing house information in reference [18] is incorrect, and the authors have made changes to it.

18. MacDicken KG, et al. A guide to monitoring carbon storage in forestry and agroforestry projects. Winrock International Institute for Agricultural Development, USA; 1997.

[33], [37], [55], [58], and [67] are Chinese papers, and the authors used (in Chinese) for annotation at the end and made modifications to the presentation of author information.

33. Wang JN, Wang WC, Hai MM. Simulation and Analysis of Land Use Change in Shandong Province Based on the PLUS Model. Territory and Natural Resources Research. 2022;(06):1–8. (in Chinese)

37. He Q. Ecosystem Service Function Assessment and Multi-Scenario Prediction of the Eastern Hubei Region Based on the InVEST Model. Hubei Normal University; 2023. (in Chinese)

55. Xu C, Ruan HH, Wu XQ, Xie YC, Yang Y. Research Progress on the Impact of Drought on Forest Soil Organic Carbon Turnover and Accumulation. Journal of Nanjing Forestry University (Natural Sciences Edition). 2022;46(6):195. (in Chinese)

58. Yang F, Zhang GL, Huang LM, Li DC, Yang F, et al. Vertical Distribution Characteristics and Influencing Factors of Soil Organic Carbon and Inorganic

Carbon in the Topographic Sequence of Alpine Mountain Regions. Acta Pedologica Sinica. 2015;52(06):1226–1236. (in Chinese)

67. Li N, Wang GX, Gao YH, Ji CZ. Research Progress on Soil Organic Carbon in the Ecosystems of the Qinghai-Tibet Plateau. Soils. 2009;41(4):512–519. (in Chinese)

The authors have made modifications to the author information representation methods in [35] and [48].

35. Zhang YL. Integration dataset of Tibet Plateau boundary; 2019. Available from: https://dx.doi.org/10.11888/Geogra.tpdc.270099.

48. Wang L, Zeng H, Zhang YJ, Zhao G, Chen N, Li JX. A review of research on soil carbon storage and its influencing factors in the Tibetan Plateau. Chinese Journal of Ecology. 2019;38(11):3506.

Reviewer1:

Comments:

The authors have adequately addressed my concerns in the previous round of review, I have no further comments and recommend the publication of this article in PLOS ONE.

Reply: The author wants to express their gratitude to the reviewer for their affirmation.

Reviewer2:

1. The article has been significantly improved in content, structure and methodology, especially in the Results and Discussion section, where many valuable analyses have been added.

2. Focus on core findings and reduce redundancy.

Reply: The authors would like to express their gratitude to the reviewer for their comment.
